# Interprofessional Field Experiences in Occupational Safety and Health

**DOI:** 10.5430/ijhe.v12n6p1

**Published:** 2023-09-11

**Authors:** Gordon L. Gillespie, Sara M. Tamsukhin, Cynthia Betcher, Tiina Reponen

**Affiliations:** 1 College of Nursing, University of Cincinnati, Cincinnati, Ohio, United States; 2 Department of Environmental and Public Health Sciences, University of Cincinnati, Cincinnati, Ohio, USA

**Keywords:** service learning, field trip, occupational health nursing, occupational safety and health engineering, industrial health, occupational medicine

## Abstract

Field trips are beneficial to students, because they provide experiences outside of the traditional classroom. Incorporating field trips into graduate programs can increase students’ exposures to real world experiences so that they can incorporate that knowledge as they complete their program. The purpose of the project was to collect and analyze graduate student feedback on 13 in-person interprofessional field trips focused on occupational safety and health. Data were collected through post-field trip structured discussions. Content analysis was used to determine themes. Five themes emerged from the data: Personal Value, Networking and Meeting, Health and Safety Planning and Policy, Environment, and Logistics and Planning. Field trips are valuable learning experiences for graduate students. The field trips in this study offered concrete experiences in occupational safety and health. Post-field trip, students provided feedback through structured discussions, which allowed for reflective observation. Overall, students found personal value in the field trips, observed health and safety procedures and policies in action, learned about various work environments, and provided input on the logistics and planning of field trips.

## 1. Introduction

### 1.1 Introduce the Problem

Out-of-classroom learning experiences have long been a staple in primary and secondary school systems (e.g., museum visit, fire department tour) (Falk, Kofan, Dierking, 1986). In higher education, out-of-classroom learning experiences include job shadowing, interviewing experts in the field, and professional field trips (Achen et al., 2019). Advantages to professional field trip experiences are the comparatively low time commitment required as well as the uniform experience for all students in a course. The purpose of the current paper is to share students’ feedback on their experiences with professional field trips and evaluate field trips as an experiential learning activity.

### 1.2 Background

Eitel explained that during field trips the “sites themselves serve as classroom, text, and subjects of inquiry” (Eitel, 2020, p. 87). Field trips allow students to have first-hand exposure to policies and practices used at a visit site (Eitel, 2020). This exposure helps students make connections to and recall course constructs (Achen et al., 2019; Eitel, 2020; Fedesco, Cavin, & Henares, 2020). Students also network with site workers, leaders, and subject matter experts (Achen et al., 2019; Eitel, 2020; Fedesco et al., 2020). The site individuals can explain constructs concretely in ways that would remain abstract in a classroom setting as well as provide a visual example of what is being discussed. Providing opportunities for students to directly ask questions to people at the visit site is particularly important (Achen et al., 2019). Professional field trips also permit students to discuss course information and the site experience in a relaxed and in-depth manner that would normally not occur within a classroom setting (Achen et al., 2019; Eitel, 2020; Fedesco et al., 2020).

Additional advantages to professional field trips are the interpersonal relationships developed between students, between students and faculty, and between students and site employees/employers. Due to increased time together and opportunities to learn about each other during casual settings (e.g., driving to field trip site together, eating meals together, sharing a hotel room during overnight trips), students increased their personal relationships (Fedesco et al., 2020). These relationships can be leveraged later while matriculating through their college program to develop study groups and complete team-based assignments together. Their future discussions for other courses can draw upon their professional field trip experiences to continue to conceptualize course constructs.

Professional field trips have been used in various higher education courses such as geology, political science, environmental studies, philosophy, and comparative literature (Fedesco et al., 2020). Fedesco et al. (2020) reported professional field trips spanning two to eight days. Some of their professional field trips included consecutive days with an overnight stay allowing them to be immersed in the cultural environment where their work was to be performed (e.g., geological field work). The current paper examines interprofessional field trips offered by one university.

### 1.3 Interprofessional Education

Students make assumptions of students from different disciplines (Gunaldo et al., 2021). Interprofessional education has the potential to rectify these assumptions by witnessing how other disciplines approach or mitigate challenges. Interprofessional education also can improve interprofessional communication, particularly among the health professions and when they follow formal communications training (Nørgaard et al., 2012).

Professional field trips can be optimized for interprofessional learning by including students from at least two disciplines. The Interprofessional Education Collaborative depicts four competencies essential for interprofessional education focused on values and ethics, roles and responsibilities, interprofessional communication, and team and teamwork (Interprofessional Education Collaborative, 2016). These core competencies were incorporated into the design and implementation of the current project. Values and ethics were incorporated using sub-competency VE4: “Respect the unique cultures, values, roles/responsibilities, and expertise of other health professions and the impact these factors can have on health outcomes” (Interprofessional Education Collaborative, 2016, p. 11). Roles and responsibilities were incorporated using sub-competency RR7: “Forge interdependent relationships with other professions within and outside of the health system to improve care and advance learning” (Interprofessional Education Collaborative, 2016, p. 12). Interprofessional communication was incorporated through sub-competency CC4: “Listen actively and encourage ideas and opinions of other team members” (Interprofessional Education Collaborative, 2016, p. 13). Team and teamwork were incorporated through sub-competency TT11: “Perform effectively on teams and in different team roles in a variety of settings” (Interprofessional Education Collaborative, 2016, p. 14).

### 1.4 Conceptual Framework

The conceptual framework guiding this evaluation project was Kolb’s (2015) experiential learning theory. There are four major constructs to this theory that represent the experiential learning cycle: concrete experience, reflective observation, abstract conceptualization, and active experimentation. Concrete experience refers to the act of doing or having an experience such as a professional field trip. Reflective observation refers to the reviewing or reflecting on the experience. Abstract conceptualization refers to learners drawing conclusions and learning from their experience. Active experimentation refers to learners planning or conducting experiments based on their experiences.

Kolb’s experiential learning cycle has been previously used within the context of professional field trips as a “concrete experience” (Stern & Powell, 2020). Stern and Powell (2020) posited that field trips could focus on concrete experiences followed by reflective observation, or they could focus on abstract conceptualization followed by active experimentation. For the use of professional field trips in our course, we focused on the concrete experience, because most students participating were still in the first year of their graduate program. However, all students had prior experience and training from having completed a relevant undergraduate program to prepare them for the concrete experience of the professional trip. Students are expected to complete the experiential learning cycle (i.e., abstract conceptualization, active experimentation) in a future course within their respective programs (e.g., occupational health nursing practicum, industrial hygiene thesis).

## 2. Method

This project used a qualitative, descriptive study design. The [name removed for anonymity] Institutional Review Board determined this project’s activities to not be research involving human subjects as defined by the U.S. Department of Health and Human Services regulations.

### 2.1 Course Information

Interprofessional Field Experiences in Occupational Safety and Health was a 1-credit interprofessional course offered every fall, spring, and summer semester. The course was open to all students matriculating in an occupational safety and health-focused program. The course used an experiential learning context to explore the intersection and practice of several occupational safety and health disciplines. Students drew upon their respective disciplines to examine occupational safety and health in a variety of private and public occupational and legislative settings. Students also explored the influence of the organizational culture and diversity of the workforce in the respective settings. The course had four student learning outcomes:

- Describe state and federal standards and recommendations for the promotion of safety and health in diverse occupational settings.
- Examine occupational safety and health practices and risks in a variety of settings.
- Identify the influence of culture on occupational safety and health practices.
- Reflect on the interprofessional field trip experience.

Extensive planning is needed to identify relevant organizations for a professional field trip (Eitel, 2020). The lead faculty for the course was responsible for the planning and for soliciting professional field trip sites. Assistance for site identification was facilitated through alumni and an External Advisory Board for the [name removed for anonymity] Education and Research Center (ERC), which oversees the various occupational safety and health programs. Primary considerations for site selection included finding sites that appeal to a variety of professional disciplines in occupational safety and health (e.g., industrial hygiene, occupational health nursing) and limiting potential safety/risks for students visiting the site. For example, when considering a professional field trip to an underground coal mine, a regional inspector with the U.S. Mine Safety and Health Administration was consulted to identify a mine with an overall positive safety rating (i.e., absence of significant citations).

### 2.2 Setting and Sample

Study procedures were coordinated through faculty members teaching at the ERC, which is supported through a grant by the U.S. National Institute for Occupational Safety and Health. The Center is comprised of five graduate educational programs: Industrial Hygiene, Occupational Health Nursing, Occupational Medicine, Occupational Safety and Health Engineering, and Biomonitoring. The program awards master of science, doctor of nursing practice, and doctor of philosophy degrees and provides occupational medicine residency.

All Center students were invited to participate in the professional field trips. Students were informed that travel expenses would be paid by a grant. The number of students participating in each field trip varied and sometimes was limited by the site host. Some students participated in more than one professional field trip. The Center provided information to collaborating Education and Research Centers in the Midwest United States and also reserved spots for other Centers’ students or faculty to join the professional field trips.

Due to the impact of COVID-19 pandemic and subsequent closures of field trip sites in the U.S. for in-person experiences, several field trips were canceled or reformatted as virtual experiences. Data from virtual experiences taking place from March 2020 through December 2021 were excluded from analysis in this report.

### 2.3 Procedures

The procedures for the professional field trips incorporated the recommendations of Stern and Powell, who advised that students receive information on what to expect during professional field trips, explore information about subject matter that will be investigated during professional field trips and participate in a discussion or complete a reflective assignment after the professional field trip is completed (Stern & Powell, 2020). Prior to the travel date, optimally a week in advance, detailed itineraries were provided to students, which included names and positions/roles for people they would be meeting. Students also had to complete assignments in order to prepare for the visit site in advance (e.g., management of air particulates in underground coal mines, risks of chemicals used in beer bottling industry). Following the professional field trips, faculty chaperone(s) facilitated a formal debriefing about students’ educational experiences. The debriefings took place during a group dinner or on the bus ride back to the university campus. The faculty asked the following questions of students:

- What did you learn during this trip?
- How do you feel about this trip?
- Why was this trip/experience valuable to you?
- Why is it important to meet with representatives from different companies?
- How can you use this field trip experience in your research and future work as an occupational safety and health specialist?

The faculty documented narrative notes of student responses. The faculty came to agreement on the validity of the notes prior to their use for analysis. Within a week of return to campus, students wrote 250–350 word blogs about their field trip experiences, which were published on the Center’s webpage.

### 2.4 Data Analysis and Trustworthiness

The qualitative debriefing data from 13 field trips were analyzed using a manifest content analysis method (Bengtsson, 2016). Initially, the data were read and re-read multiple times to gain a sense of their meaning. Next, meaning units were identified that reflected the debriefing questions. The meaning units were condensed to codes representing clusters of meaning units. The codes then were extrapolated to themes representing the composite data.

Trustworthiness of the data was based on guidance from Lincoln and Guba (1985). First, credibility of the data was accomplished using two strategies: data triangulation and investigator triangulation. Data triangulation was used by collecting data from multiple field trip experiences (n = 13) versus a single experience (n = 1). Investigator triangulation was used by having two investigators independently analyze the data and come to agreement on the study findings. Second, dependability of the data was accomplished through the development of the coding schema. Although the investigators conducted their analyses independently, they met during milestone events (i.e., initial development of coding schema, finalization of coding schema) to verify that subsequent analyses would align.

## 3. Results

Graduate students participated in field trips across multiple industries including U.S. federal agencies, mining, and manufacturing. The number of students per trip varied from 5 to 17. Students were chaperoned by 1 to 3 faculty members per trip. See Table for additional information about field trip locations, industries, dates, number of attendees, and lengths of trips.

Five themes were identified through the data analysis: Personal Value, Networking and Meeting, Health and Safety Planning and Policy, Environment, and Logistics and Planning. The Personal Value theme was defined as participants’ statements of the value they gained from the field trip experience. The Networking and Meeting theme reflected those connections made by the students during the field trips. The theme of Health and Safety Planning and Policy included any mentions of safety or health-related features noted at the field trip sites. The Environment refers to any description of the physical environment related to the senses; for example, what students could hear, touch, or see. The final theme was Logisitics and Planning. This theme included any student mentions of the positives or shortcomings of the planning or logistics of the field trips.

### 3.1 Personal Value

Students reported a “Personal Value” for attending the field trip, which were categorized into five sub-themes: Academic and Research Environment, Seeing Things in Practice, Interprofessional Practice, Business Environment, and General Value. Personal value for an “Academic and Research Environment” was identifying possible research topics and data sources, and the need for further education. For example, students saw potential to access sites for employees and employment data, focus research on work-related regulations, and the need to learn toxicology.

**Figure 1.**
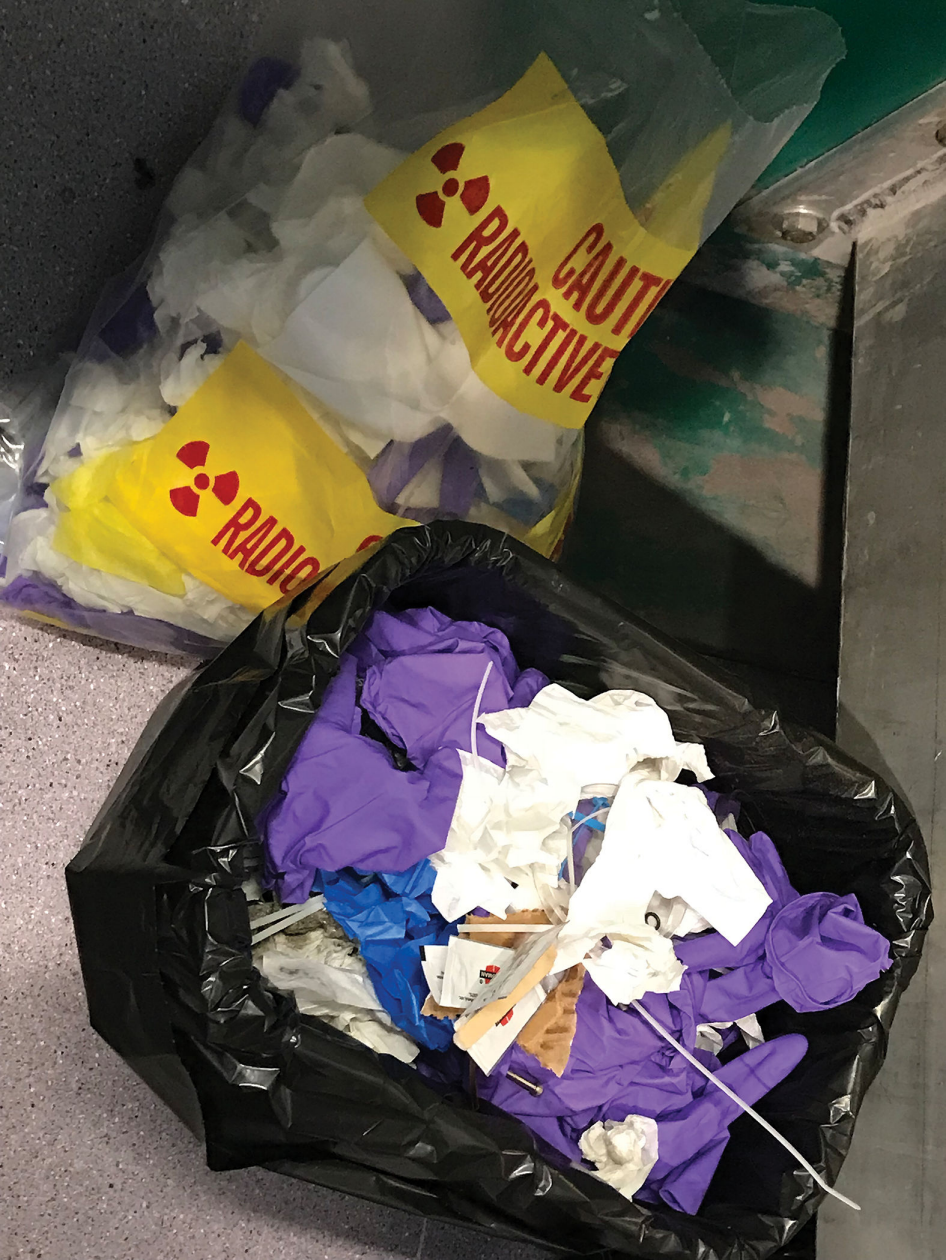
Disposal products including regular trash and potentially radioactive waste.

In their feedback, students also described “Seeing Things in Practice”, reporting specific knowledge about the business environment of each field trip location. The field trips were an opportunity to learn more about specific companies and their operations through first-hand experience and the role of occupational safety and health experts within companies. Students observed several workers doing repetitive tasks and not switching roles, telehealth in use, advances in dust and noise prevention and mitigation, and management of waste (see Figure 1).

“Interprofessional Practice” was observed by students during interactions between multiple disciplines. In the interviews, participants identified interactions between disciplines and how these contributed to the ‘big picture.’ Participants noted that these interdisciplinary interactions were successful. For example, a health physicist was observed overseeing maintenance in the high risk area of a flux reactor; something students had only read about in books.

The “Business Environment” was both about showing the benefit of occupational safety and health specialists and translating knowledge to practice. For example, students discussed a continual need to make the “business case” for their services. They also reported the benefit of how the experiences would help them consult with companies from a variety of discplines and incorporate solutions from one business into another.

**Figure 2.**
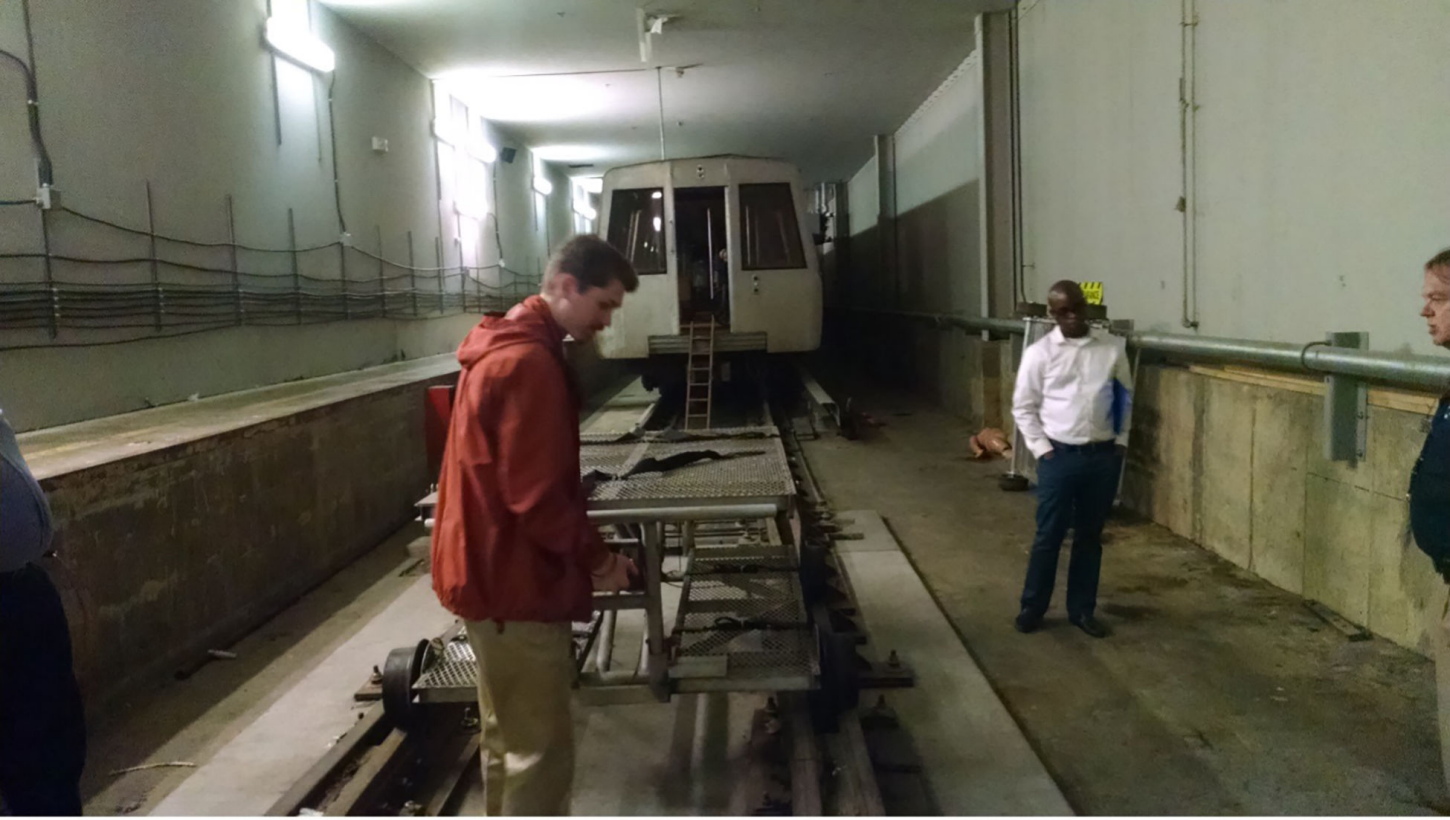
Students discussing mass casualty evacuation in a subway tunnel training center.

Students also commented on the “General Value” of the field trip experiences. For example, they were able to get close to equipment they only heard about (see Figure 2), seeing the relationship between businesses and the communities in which they were situated, and thinking about problems more holistically.

### 3.2 Networking and Mentoring

“Networking and Meeting” focused on the additional aspects inherent with field trip experiences. Students had opportunities to network and meet with occupational safety and health specialists, industry leaders, government employees/officials, legislators, and program alum. Interactions took place during formal presentations, site tours, and meals (e.g., lunch, dinner). Students leveraged the interactions to not only connect with and learn from leaders in the field, they were able to identify internship and employment possibilities.

### 3.3 Health and Safety Planning and Policy

“Health and Safety Planning and Policy” encompassed activities ranging from the local to national level. Locally, occupational health planning and policy focused on health promotion programs, prevention and mitigation interventions, and policies to prevent injuries. For example, occupational sites used proximity sensors to prevent injury from mining equipment, conducted annual screening programs, and posted signage about number of injury free days. At the national level, students explored the difference between roles and function of the U.S. Occupational Safety and Health Administration (OSHA) and U.S. National Institute for Occupational Safety and Health (NIOSH), process to pass federal law and establish occupational health standards, and how standards are monitored and enforced in occupational settings.

### 3.4 Environment

**Figure 3.**
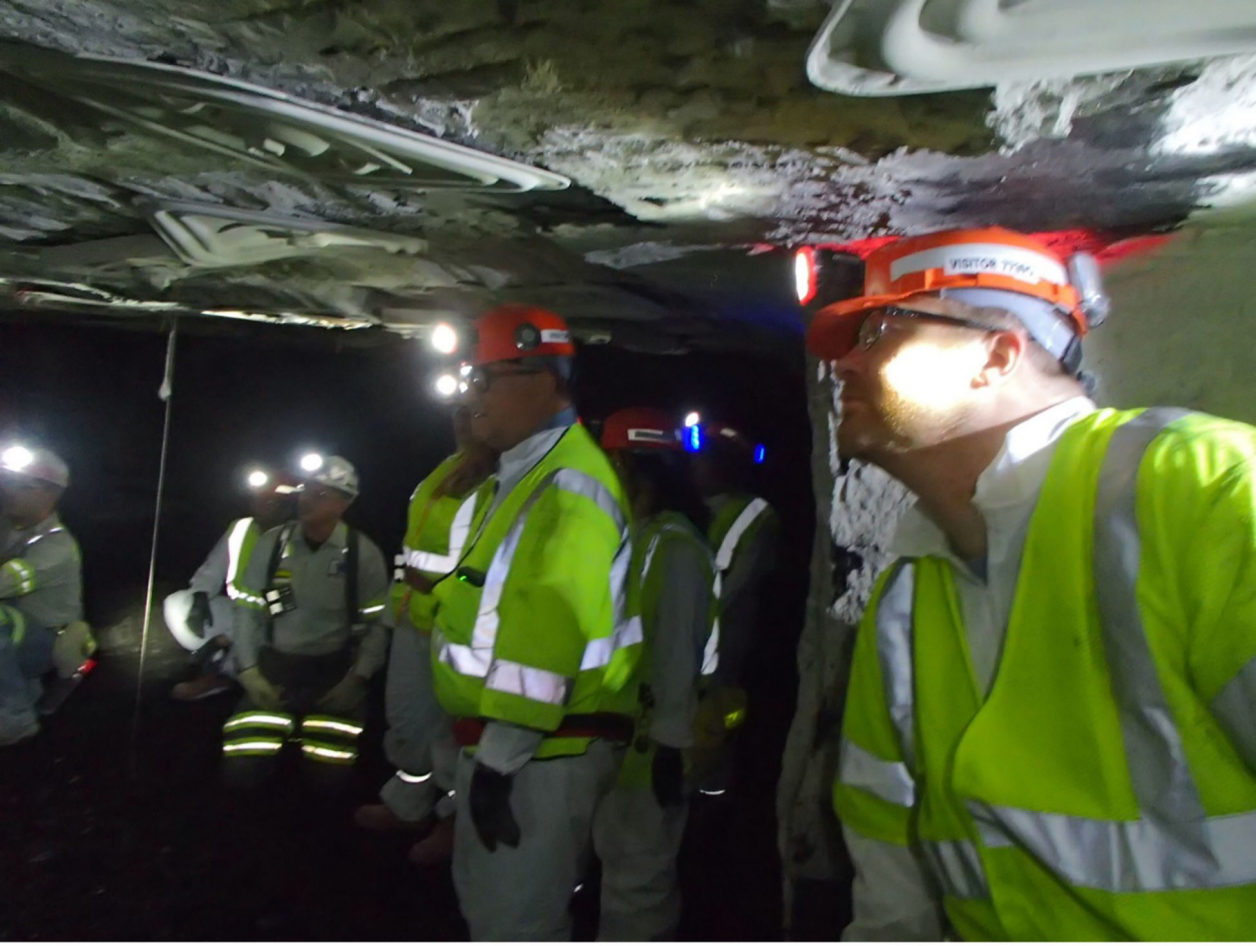
Students touring an underground coal mine with notable low ceilings, support structures attached to the ceiling, and limestone applied to surfaces.

”Environment” related to the physical environments of the sites, which included visual observations as well as what they heard or smelled. At a manufacturing plant, students noted a roof top garden with flowers. While in an underground coal mine, students noticed that it was not as noisy as they thought it would be. Another student observed that the walls of the coal mine were sprayed with limestone and the mine was cleaner than they had imagined (see Figure 3). At the waste management site, students stood inside the control room for the energy plant that was converting methane gas produced from decomposing solid waste into energy to power a nearby urban city. Inside the control room, they also felt the vibrations from the noise being buffered in the adjacent room.

### 3.5 Logistics and Planning

“Logistics and Planning” conveyed students’ perceptions about the adequacy for trip planning, itinerary, meals, lodging, and related aspects of the field trips. Although one student from an early trip believed that there was no clear purpose to the experience, a student from a subsequent trip commented about that trip having a very organized itinerary. During the trip to Washington, DC, students commented that the visit was very busy with no free time. Students also commented about limited food options for hotel breakfasts as well as commenting about loving the food at other locations. Overall, students believed the employees at each site were welcoming and hospitable. Another aspect to the field trip experiences commented on by students was that they wished the field trips had aligned more closely to their program (i.e., specifically the occupational safety and health engineering students). Other students commented on how they learned more about the other occupational safety and health disciplines from within the Center.

## 4. Discussion

Field trips offer students an opportunity to physically experience and engage with content that they learn in classrooms. In fact, field trips themselves serve as a classroom (Eitel, 2020). These experiences were further enhanced through their use of an interpofessional lens—the inclusion of students from multiple occupational health disciplines (McCullagh et al., 2022). When students visit occupational health sites (e.g., plants, federal agencies), they can gain first-hand exposure to policies and practices focused on occupational safety and health prevention (Eitel, 2020). For this project, students had the opportunity to participate in field trips through the Interprofessional Field Experiences in Occupational Safety and Health course. Based on the overall evaluation of the cumulative field trips, the experiences were positive and yielded significant experiential learning by the students with high potential to continue positively impacting their education and future internships and employment.

Kolb’s experiential learning theory provided a framework for the field trips in the current project (Kolb, 2015). This learning theory included four parts: concrete experience, reflective observation, abstract conceptualization, and active experimentation. The first construct, concrete experiences, was incorporated through concrete learning experiences in occupational safety and health. Following the field trip experiences, students provided feedback through structured discussions. Students connected the experiences from field trips to the materials they learned in the classroom. The interprofessional experiences also allowed them to reflect on and correct their assumptions about the other disciplines and what those disciplinary professionals do in actual practice (McCullagh et al., 2022). Eitel and Shaby, Assaraf, and Koch suggest several ways that students can connect field trips to a course including projects, logs, presentations, and papers (Eitel, 2020; Shaby, Assaraf, & Koch, 2023). For the present study, students wrote 250–350 word blogs after returning from their field trips. This allowed the students to document their learning. The blog data, however, were not analyzed for the present study.

Reflective observation, the second construct in Kolb’s experiential learning theory, was met through structured discussions following the field trips (Kolb, 2015). The discussions served a dual purpose—collect student feedback and incorporate reflective observation. Analysis of this feedback identified five themes: Personal Value, Networking and Meeting, Health and Safety Planning and Policy, Environment, and Logistics and Planning.

Field trips provide benefits to students and the profession. Field trip locations should relate to students’ professional interests (Eitel, 2020). Achen et al. noted that networking and professional development also were results of field trips (Achen et al., 2019). Student feedback in the present project indicated that students valued the field trip experiences and were an opportunity to observe potential careers and employers in occupational safety and health. Additionally, students learned about the various corporations and could network with employees. Students identified field trips as a resource for additional education, research, and employment.

While students identified personal benefits of field trips, they also noticed details about the health and safety operations at each location. Students observed their health and safety procedures. Students also reflected on the work environment and safety culture as well as career paths of the safety and operation’s managers at each field trip location. Again, field trips provided concrete experiences that would not be possible within the traditional classroom setting. Rokooei and Rokooei similarly noted that moving education out of the classroom and into a real world environment allows students to see the practical challenges of a discipline, interact with professionals who can discuss mitigation strategies for these challenges, and network with professionals in advance of applying for employment at a field trip site (Rokooei & Rokooei, 2021).

Field trips are beneficial to students but require planning. Eitel emphasized the need for planning and pre-arranging activities (Eitel, 2020). In the present study, students provided both positive and negative feedback on the logistics and planning of the field trips. From this feedback, faculty were able to determine student needs and preferences to ensure that future field trips are meaningful to them.

### 5.1 Limitations

The present study reviewed the feedback from students following 13 field trips. The data were collected during structured discussions post-field trip. The faculty conducted these discussions either on the bus or during meals while the experience was still fresh. However, due to logistics, the discussions were not recorded verbatim, which would have strengthened the data and findings of this report. Although faculty took detailed notes, it is possible that faculty misheard information from students. This limitation was mitigated in part by two faculty chaperones noting the same student comments. In addition, due to the qualitative nature of the study and source of data, the findings are not generalizable.

### 5.2 Conclusion

Field trips are valuable learning experiences for graduate students. The field trips in this study offered concrete experiences in occupational safety and health. Post-field trip, students provided feedback through structured discussions, which allowed for reflective observation. Overall, students found personal value in the field trips, observed health and safety procedures and policies in action, learned about various work environments, and provided input on the logistics and planning of field trips. Future field trips can incorporate the remaining constructs of Kolb’s experiential learning theory—active conceptualization and active experimentation—to yield even higher learning outcomes.

## Figures and Tables

**Table 1.** Field trip experiential sites organized by occupational safety and health focus.

Focus and site information	Date	Students (n)	Faculty (n)	Length of trip

*Health Policy and Transportation Industry* (Congressional staff, U.S. Environmental Protection Agency, U.S. National Institute for Occupational Safety and Health, U.S. Occupational Safety and Health Administration, Washington Metropolitan Area Transit Authority, Teamsters)	March 2016	7	3^a^	3 days
*Innovation, Renewable Energy, Environment* (Purdue University, nanotechnology center, wind farm, anaerobic digester, landfill)	August 2016	7	1	2 days
*Environment/Manufacturing* (water reclamation plant, beer manufacturing and bottling plant)	August 2017	5	1	2 days
*Innovation*				
Argonne National Laboratory	May 2018	11^b^	2	2 days
Oak Ridge National Laboratory	August 2018	15	2	2 days
*Manufacturing*				
Automotive manufacturing plant	March 2017	8	3	1 day
Chemical manufacturing plant	March 2019	8^c^	2	2 days
Petroleum manufacturing plant	May 2019	6	1	2 days
Beer manufacturing and bottling plant	January 2020	6	1	1 day
*Mining Industry*				
Underground coal mine	May 2017	8	2	2 days
Underground coal mine	August 2019	17	2	2 days
Underground coal mine	March 2022	8	1	2 days
*Transportation Industry*				
River barge company	August 2022	6	2	2 days

a 1 faculty was from a partner university

b 4 students were from a partner university

c 1 student was from a partner university
